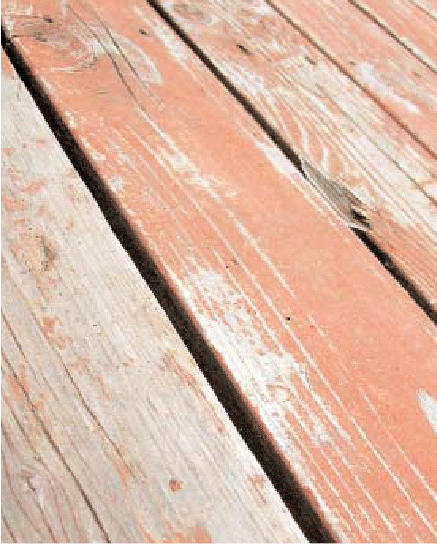# The Beat

**Published:** 2006-05

**Authors:** Erin E. Dooley

## Easy Rider, Easy Polluter

A Swiss study published in the 1 January 2006 issue of *Environmental Science & Technology* shows that motorcycles collectively emit 16 times more hydrocarbons, 3 times more carbon monoxide, and “disproportionately high” levels of other air pollutants, compared with passenger cars. Two- and three-wheeled vehicles are widely used in Asia. Because they are not a primary means of transportation in developed countries, however, not a great deal of attentionhas been paid to emissions from these vehicles. But a U.S. EPA rule that took effect in January 2006 requires manufacturers to reduce emissions of hydrocarbons and nitrogen oxides by 60%. By 2010 the EPA estimates the rule will save about 54,000 tons of emissions and 12 million gallons of fuel per year.

**Figure:**
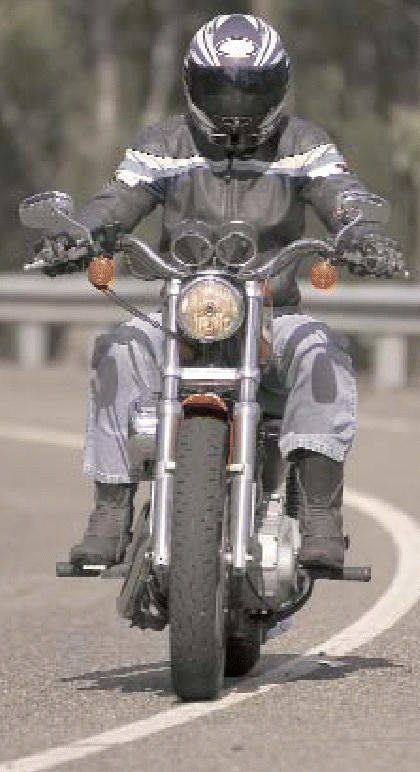


## Beverages Doing Better

Last year, the EU commissioner for health and consumer affairs called on drink and food companies to take steps to fight the growing problem of child obesity. In response, the Union of European Beverages Associations (UNESDA) announced in January 2006 that it would limit advertising targeted at youth, control sales in schools, and improve nutritional labels. It further agreed to provide drinks, including sugar-free and low-calorie options, in smaller container sizes to limit intake. Also, vending machines in schools will carry images of a healthy, active lifestyle and a balanced diet, rather than brand logos. Global drink firms including The Coca-Cola Company and Cadbury Schweppes European Beverages are members of UNESDA.

## Score for the Environment

In November 2005, sporting goods manufacturers from Sialkot, Pakistan, who produce 60% of the world’s soccer balls, pledged to reduce and improve the use of water and energy during their manufacturing processes. They also agreed to introduce cleaner technology, reduce toxic wastes, and raise environmental awareness among their workers. This agreement was part of the Third Global Forum for Sports and Environment, which brought together more than 200 participants from the world of sports and sporting goods manufacturing to discuss their impact on and contribution to sustainable development. In 1997 this group of manufacturers took a big step by eliminating child labor in the Sialkot soccer ball industry.

**Figure:**
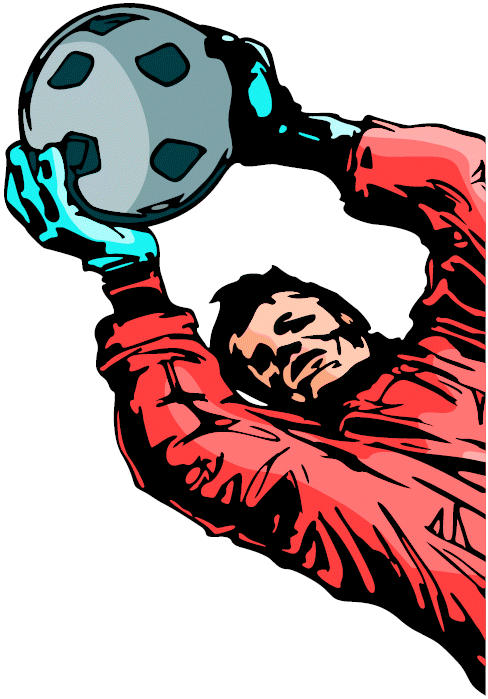


## A “Cowabunga!” Moment for Farmers

Penn State researchers have come up with a cheaper, safer way to clean and disinfect milking equipment. Conventional cleaning systems use expensive acids and chlorinated chemicals that can burn the eyes and skin and damage the environment. The new process uses electrolyzed oxidizing water, produced when electric current flows through two electrodes immersed in a weak saline solution and separated by a membrane. Tests showed that the electrolyzed oxidizing water was as effective as conventional treatments at removing organic matter from a series of pipes set up to simulate real milking equipment. Electrolyzed oxidizing water is also effective for cleaning other agricultural products such as fresh produce and eggs.

**Figure:**
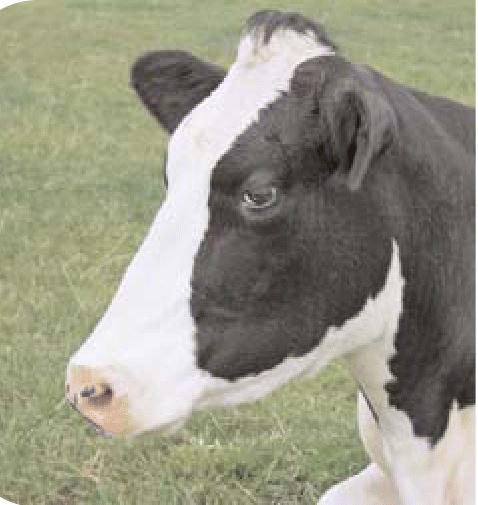


## Of Minors and Miners

Ghana’s Institute of Journalism is objecting to a public relations campaign in a weekly children’s newspaper, *Junior Graphic*, that focuses on promoting positive information about the gold mining industry. The campaign is funded by the mining company Newmont Ghana. The journalists decry the fact that the campaign targets children, and question its timing, months after the company was accused of knowingly dumping human waste into a river that provides drinking water for local communities. The Denver, Colorado–based mining company is the world’s largest gold mining organization.

## Arsenic and Old Decks

Two papers published by Florida researchers in the 1 February 2006 issue of *Environmental Science & Technology* highlight the threat posed by arsenic from treated lumber used in decks, utility poles, and fences. Though chromated copper arsenate (CCA)–treated wood was phased out of residential use in 2003, arsenic from wood already in use will likely leach into the environment for years to come, possibly threatening groundwater. One of the papers estimated that of 28,000 tons of arsenic used in Florida as of 2000 for CCA-treated wood, 5,000 tons had already leached to underlying soils. The paper added that over 12,000 more tons will leach from structures by 2040. Currently Florida law does not require that construction and demolition landfills be equipped with linings.

**Figure:**